# Supplementary material for: Potential economic and clinical implications of improving access to snake antivenom in five ASEAN countries: A cost-effectiveness analysis
Source: PLoS Negl Trop Dis. 2022 Nov 16;16(11):e0010915. doi: 10.1371/journal.pntd.0010915 (PMC9668136; doi:10.1371/journal.pntd.0010915)
Supplement: S1 Table — (DOCX) [file pntd.0010915.s002.docx]

**S1 Table** Input parameters for economic evaluation of improving access to snake antivenom in ASEAN countries

| Parameters (Distribution) | Indonesia | Philippines | Vietnam | Lao PDR | Myanmar |
| --- | --- | --- | --- | --- | --- |
| Epidemiological parameters |  |  |  |  |  |
| Total population, million people[1] | 271 | 108 | 96 | 7 | 54 |
| Incidence of snakebite per 100,000 population (Beta)[2-4] | 49.9  (49.6 to 50.2) | 12.4  (10.5 to 14.5) | 48.5  (19.5 to 99.8) | 200.0  (196.7 to 203.3) | 39.0  (38.4 to 39.5) |
| Probability of snakebite victims seeking conventional treatment only in current access (Beta)[3] | 0.75 | 0.00 | 0.00 | 0.00 | 0.34  (0.32 to 0.35) |
| Probability of snakebite victims firstly seeking traditional treatment then switching to conventional treatment in current access (Beta)[3, 4] | 0.00 | 0.27 | 0.43  (0.29 to 0.59) | 0.10 | 0.07  (0.05 to 0.08) |
| Probability of snakebite victims firstly seeking traditional treatment then switching to conventional treatment in full access | 1.00 | 1.00 | 1.00 | 1.00 | 1.00 |
| Probability of systemic envenoming indicated for antivenom treatment for victims seeking conventional treatment (Beta)[3-6] | 0.40  (0.26 to 0.57) | 0.16  (0.12 to 0.21) | 0.90  (0.87 to 0.93) | 0.27  (0.20 to 0.35) | 0.79  (0.76 to 0.81) |
| Probability of systemic envenoming for victims seeking traditional treatment | 0.26 | 0.12 | 0.87 | 0.20 | 0.76 |
| Probability of victims indicated for antivenom treatment regardless of treatment seeking behavior who received antivenom treatment at current level of access* | 0.10 | 0.26 | 0.37 | 0.04 | 0.64 |
| Probability of antivenom given to victims with systemic envenoming seeking care at the healthcare facilities at current level of access* | 0.12 | 0.77 | 0.83 | 0.33 | 1.00 |
| Probability of antivenom given to victims with systemic envenoming seeking care at the healthcare facilities at full level of access | 1.00 | 1.00 | 1.00 | 1.00 | 1.00 |
| Probability of adverse reaction following antivenom treatment (Beta)[3, 6, 7] | 0.40 | 0.05 | 0.22  (0.03 to 0.60) | 0.53  (0.38 to 0.69) | 0.08  (0.06 to 0.10) |
| Probability of death in snakebite victims without systemic envenoming[2, 3, 5, 6] | 0.00 | 0.00 | 0.00 | 0.00 | 0.00 |
| Probability of death in systemic envenoming treated with antivenom (Beta)[3, 5, 6, 8] | 0.09  (0.07 to 0.12) | 0.07  (0.01 to 0.18) | 0.015  (0.008 to 0.025) | 0.05  (0.01 to 0.16) | 0.068  (0.061 to 0.074) |
| Relative risk of death when antivenoms are not available (Log-normal)[8] | 2.33  (1.26 to 4.06) | 2.33  (1.26 to 4.06) | 2.33  (1.26 to 4.06) | 2.33  (1.26 to 4.06) | 2.33  (1.26 to 4.06) |
| Probability of death in systemic envenoming treated in hospital without antivenom (Beta) | 0.21 | 0.16 | 0.035 | 0.11 | 0.16 |
| Probability of death in systemic envenoming not treated in hospital (Beta) | 0.28 | 0.43 | 0.057 | 0.37 | 0.17 |
| Probability of digit amputation due to snakebite envenoming (Beta)[3, 4, 6] | 0.01  (0.005 to 0.02) | 0.007 | 0.00 | 0.05  (0.01 to 0.16) | 0.00 |
| Probability of limb amputation due to snakebite envenoming (Beta)[3] | 0.01  (0.005 to 0.02) | 0.003 | 0.00 | 0.02  (0.001 to 0.12) | 0.00 |
| Disability weight |  |  |  |  |  |
| Disability weight for victims not indicated for antivenom treatment (Beta)[9] | 0.006  (0.002 to 0.012) | 0.006  (0.002 to 0.012) | 0.006  (0.002 to 0.012) | 0.006  (0.002 to 0.012) | 0.006  (0.002 to 0.012) |
| Disability weight for victims indicated for antivenom treatment (Beta)[9] | 0.163  (0.109 to 0.227) | 0.163  (0.109 to 0.227) | 0.163  (0.109 to 0.227) | 0.163  (0.109 to 0.227) | 0.163  (0.109 to 0.227) |
| Disability weight for digit amputation (Beta)[9] | 0.005  (0.002 to 0.010) | 0.005  (0.002 to 0.010) | 0.005  (0.002 to 0.010) | 0.005  (0.002 to 0.010) | 0.005  (0.002 to 0.010) |
| Disability weight for limb amputation (Beta)[9] | 0.039  (0.024 to 0.059) | 0.039  (0.024 to 0.059) | 0.039  (0.024 to 0.059) | 0.039  (0.024 to 0.059) | 0.039  (0.024 to 0.059) |
| Duration of disease |  |  |  |  |  |
| Length of stay for victims not indicated for antivenom treatment, day | 1 | 1 | 1 | 1 | 1 |
| Length of stay for victims indicated for antivenom treatment (Gamma)[10] | 6.1 (4.1 to 8.0) | 6.1 (4.1 to 8.0) | 6.1 (4.1 to 8.0) | 6.1 (4.1 to 8.0) | 6.1 (4.1 to 8.0) |
| Unit costs, USD |  |  |  |  |  |
| Unit cost of hospitalization for victims not indicated for antivenom treatment (Gamma)[11-15] | 175  (158 to 193) | 68  (61 to 75) | 31  (28 to 34) | 69  (62 to 76) | 36  (32 to 39) |
| Unit cost of hospitalization for victims not indicated for antivenom treatment (Gamma)[11-15] | 1,007  (907 to 1,108) | 408  (367 to 449) | 173  (155 to 190) | 372  (335 to 410) | 210  (189 to 231) |
| Unit cost of antivenom treatment (Gamma)[11, 13] | 773  (695 to 850) | 312  (281 to 343) | 68  (61 to 75) | 202  (181 to 222) | 326  (293 to 359) |
| Unit cost of antivenom logistics costs, percentage of antivenom price[16] | 0.05 | 0.05 | 0.05 | 0.05 | 0.05 |
| Unit cost of adverse reaction management (Gamma)[11, 13] | 3.8 (3.5 to 4.2) | 4.4 (4.0 to 4.9) | 5.1 (4.6 to 5.7) | 5.1 (4.6 to 5.7) | 0.76 (0.69 to 0.84) |
| Unit cost of digit amputation (Gamma)[16] | 96 (86 to 106) | 96 (86 to 106) | 96 (86 to 106) | 52 (47 to 57) | 96 (86 to 106) |
| Unit cost of limb amputation (Gamma)[16] | 154 (138 to 169) | 154 (138 to 169) | 154 (138 to 169) | 158 (143 to 174) | 154 (138 to 169) |
| Unit cost of traditional healer | 0 | 0 | 0 | 0 | 0 |
| Unit cost of transportation (Gamma)[17] | 7.8 (7.0 to 8.6) | 5.8 (5.2 to 6.4) | 21 (19 to 23) | 4.7 (4.2 to 5.1) | 20 (18 to 22) |
| Unit cost of additional food (Gamma)[18] | 2.3 (2.1 to 2.6) | 2.3 (2.1 to 2.6) | 6.5 (5.9 to 7.2) | 2.4 (2.3 to 2.5) | 3.3 (3.0 to 3.6) |
| Others |  |  |  |  |  |
| Discount rate[19, 20] | 0.03  (0.00 to 0.06) | 0.03  (0.00 to 0.06) | 0.03  (0.00 to 0.06) | 0.03  (0.00 to 0.06) | 0.03  (0.00 to 0.06) |
| GDP per capita, USD[21] | 4,136 | 3,485 | 2,715 | 2,625 | 1,421 |
| GDP per capita annual growth, %[22] | 0.04 | 0.05 | 0.06 | 0.03 | 0.02 |
| Number of relatives or family members who companied snakebite victims | 1 | 2 | 1 | 1 | 2 |

Parameters are presented as base-case value (range). Costs are presented as USD where 1 USD equals to 14,147.67 Indonesian Rupees, 51.80 Philippine Pesos, 23,050.24 Vietnamese Dongs, 8,679.41 Lao Kips, and 1,518.26 Myanmar Kyats. *– Probability of antivenom given to victims with systemic envenoming seeking care at the healthcare facilities was determined by the number of antivenom treatments available divided by the number of snakebite victims needed antivenom treatment who sought care at the healthcare facilities.

**References**

1. World Bank. Population, total [Internet]. 2019 [cited 2021 Apr 13]. Available from: <https://data.worldbank.org/indicator/SP.POP.TOTL>.

2. Watt G, Padre L, Tuazon ML, et al. Bites by the Philippine cobra (Naja naja philippinensis): an important cause of death among rice farmers. *Am J Trop Med Hyg* 1987;37(3):636-39.

3. Mahmood MA, Halliday D, Cumming R, et al. Snakebite incidence in two townships in Mandalay Division, Myanmar. *PLoS Negl Trop Dis* 2018;12(7):e0006643.

4. Thang VV, Bao TQQ, Tuyen HD, et al. Incidence of snakebites in Can Tho Municipality, Mekong Delta, South Vietnam—Evaluation of the responsible snake species and treatment of snakebite envenoming. *PLoS Negl Trop Dis* 2020;14(6):e0008430.

5. Adiwinata R, Nelwan EJ. Snakebite in Indonesia. *Acta Med Indones* 2015;47(4):358-65. [published Online First: 2016/03/05]

6. Vongphoumy I, Chanthilat P, Vilayvong P, et al. Prospective, consecutive case series of 158 snakebite patients treated at Savannakhet provincial hospital, Lao People's Democratic Republic with high incidence of anaphylactic shock to horse derived F (ab') 2 antivenom. *Toxicon* 2016;117:13-21.

7. Le Khac Q. Clinical evaluation of snakebites in Vietnam: A study from Cho Ray hospital. 2004

8. Habib AG, Warrell DA. Antivenom therapy of carpet viper (Echis ocellatus) envenoming: effectiveness and strategies for delivery in West Africa. *Toxicon* 2013;69:82-89.

9. Salomon JA, Haagsma JA, Davis A, et al. Disability weights for the Global Burden of Disease 2013 study. *The Lancet Global Health* 2015;3(11):e712-e23.

10. Shafie NA, Fauzi H, Wahab M, et al. The prevalence of hypersensitivity reactions to snake antivenoms administered in sultanah nur zahirah hospital from 2013 to 2016. *Med J Malaysia* 2020;75(3):217.

11. Menteri Kesehatan Republik Indonesia. Peraturan Menteri Kesehatan Republik Indonesia Nomor 63 Tahun 2014 tentang Pengadaan Obat Berdasarkan Katalog Elektronik (E-catalogue). 2014

12. Edillo FE, Halasa YA, Largo FM, et al. Economic cost and burden of dengue in the Philippines. *Am J Trop Med Hyg* 2015;92(2):360-66.

13. Department of Health, Republic of the Philippines. Drug Price Reference Index [Internet]. [Available from: <https://dpri.doh.gov.ph/index.php?page=search> accessed May 10 2021.

14. Maternal and Reproductive Health Division, Department of Public Health Myanmar. Costed implementation plan to meet family planning 2020 commitments of Myanmar. Strategic prioritization of implementation 2018-2020 [Internet]. 2018 [cited 2021 May 10]. Available from: <https://www.familyplanning2020.org/sites/default/files/myanmar_cip_2018.10.pdf>.

15. Flessa S, Dung NT. Costing of services of Vietnamese hospitals: identifying costs in one central, two provincial and two district hospitals using a standard methodology. *The international journal of health planning and management* 2004;19(1):63-77.

16. Riewpaiboon A. Standard cost lists for health economic evaluation in Thailand. *Journal of the Medical Association of Thailand= Chotmaihet Thangphaet* 2014;97:S127-34.

17. Riewpaiboon A. Economic burden of hand, foot, and mouth disease in Vietnam; An evidence for priority setting and efficiency management.

18. Vodicka E, Zimmermann M, Lopez AL, et al. Japanese encephalitis vaccination in the Philippines: A cost-effectiveness analysis comparing alternative delivery strategies. *Vaccine* 2020;38(13):2833-40.

19. Teerawattananon Y. Guidelines for health technology assessment in Thailand (second edition). J Med Assoc Thai. 2014;97(5):S4–9.

20. Indonesian Health Technology Assessment Committee MoHotRoI. Health Technology Assessment (HTA) guideline.

21. World Bank. GDP per capita (current LCU) [Internet]. 2019 [cited 2021 Apr 16]. Available from: <https://data.worldbank.org/indicator/NY.GDP.PCAP.CN>.

22. World Bank. GDP per capita growth (annual %) [Internet]. 2019 [cited 2021 Apr 16]. Available from: <https://data.worldbank.org/indicator/NY.GDP.PCAP.KD.ZG>.
