# Supplementary material for: Potential economic and clinical implications of improving access to snake antivenom in five ASEAN countries: A cost-effectiveness analysis
Source: PLoS Negl Trop Dis. 2022 Nov 16;16(11):e0010915. doi: 10.1371/journal.pntd.0010915 (PMC9668136; doi:10.1371/journal.pntd.0010915)
Supplement: S3 Table — (DOCX) [file pntd.0010915.s004.docx]

**S3 Table** Scenario analyses of cost-utility analysis of improving access to snake antivenom in ASEAN countries

|  | Incremental costs per disability-adjusted life year averted, USD | | | | |
| --- | --- | --- | --- | --- | --- |
| Scenario | **Indonesia** | **Philippines** | **Vietnam** | **Lao PDR** | **Myanmar** |
| Base-case | -6,991 | -5,987 | -6,062 | -3,213 | -1,165 |
| Antivenom was effective on reducing risk of amputation following snakebite envenoming | -6,978 | -5,985 | N/A | -3,207 | N/A |
| Incorporating post-traumatic stress disorder as disability following snakebite envenoming | -7,023 | -6,015 | -6,091 | -3,228 | -1,170 |
| Excluding indirect costs | 338 | 169 | 356 | 61 | 283 |
| Logistic costs as 10% of antivenom price | -6,978 | -5,983 | -6,059 | -3,212 | -1,159 |

Willingness-to-pay thresholds of each ASEAN country were 4,136 USD for Indonesia, 2,317 USD for Philippines, 2,715 USD for Vietnam, 2,625 USD for Lao PDR, and 1,421 USD for Myanmar. Costs are presented as 2019 USD where 1 USD = 14,147.67 = Indonesian Rupees = 51.80 = Philippine Pesos = 23,050.24 Vietnamese Dong = 8,679.41 Lao Kip = 1,518.26 Myanmar Kyat. USD – US Dollars; N/A – not applicable.
