# Supplementary material for: Potential economic and clinical implications of improving access to snake antivenom in five ASEAN countries: A cost-effectiveness analysis
Source: PLoS Negl Trop Dis. 2022 Nov 16;16(11):e0010915. doi: 10.1371/journal.pntd.0010915 (PMC9668136; doi:10.1371/journal.pntd.0010915)
Supplement: S4 Table — (DOCX) [file pntd.0010915.s005.docx]

**S4 Table** Threshold analyses of antivenom effectiveness and costs of antivenom treatment resulted in an incremental cost-effectiveness ratio of 0

| Parameter | Indonesia | Philippines | Vietnam | Lao PDR | Myanmar |
| --- | --- | --- | --- | --- | --- |
| Antivenom effectiveness, Relative risk of death | | | | | |
| Base-case value | 2.33 | 2.33 | 2.33 | 2.33 | 2.33 |
| Threshold value resulting in ICER of 0 | 1.01 | 0.44 | 0.73 | 0.35 | 1.19 |
| Costs of antivenom treatment, USD |  |  |  |  |  |
| Base-case value | 773 | 312 | 68 | 202 | 326 |
| Threshold value resulting in ICER of 0 | 22,985 | 46,054 | 5,624 | 22,365 | 3,083 |
| Ratio of threshold value to base-case value | 30 | 149 | 87 | 113 | 9 |

Costs are presented as 2019 USD where 1 USD = 14,147.67 = Indonesian Rupees = 51.80 = Philippine Pesos = 23,050.24 Vietnamese Dong = 8,679.41 Lao Kip = 1,518.26 Myanmar Kyat. DALY – disability-adjusted life year; ICER – incremental cost-effectiveness ratio; USD – US Dollars.
