# Supplementary material for: Potential economic and clinical implications of improving access to snake antivenom in five ASEAN countries: A cost-effectiveness analysis
Source: PLoS Negl Trop Dis. 2022 Nov 16;16(11):e0010915. doi: 10.1371/journal.pntd.0010915 (PMC9668136; doi:10.1371/journal.pntd.0010915)
Supplement: S2 Fig — (DOCX) [file pntd.0010915.s007.docx]

**S2 Figure** One-way sensitivity analysis of incremental costs per disability-adjusted life year (DALY) averted of improving access to snake antivenom in ASEAN countries
