# Supplementary material for: Potential economic and clinical implications of improving access to snake antivenom in five ASEAN countries: A cost-effectiveness analysis
Source: PLoS Negl Trop Dis. 2022 Nov 16;16(11):e0010915. doi: 10.1371/journal.pntd.0010915 (PMC9668136; doi:10.1371/journal.pntd.0010915)
Supplement: S3 Fig — Incremental costs and deaths averted of full access to snake antivenom in each ASEAN country based on a probabilistic sensitivity analysis of 1,000 iterations are presented in dots. Willingness-to-pay thresholds of each country are presented as dash lines with corresponding color. (DOCX) [file pntd.0010915.s008.docx]

**S3 Figure** Cost-effectiveness plane of incremental costs per death averted of improving access to snake antivenom in ASEAN countries

Incremental costs and deaths averted of full access to snake antivenom in each ASEAN country based on a probabilistic sensitivity analysis of 1,000 iterations are presented in dots. Willingness-to-pay thresholds of each country are presented as dash lines with corresponding color.
