# Supplementary material for: Potential economic and clinical implications of improving access to snake antivenom in five ASEAN countries: A cost-effectiveness analysis
Source: PLoS Negl Trop Dis. 2022 Nov 16;16(11):e0010915. doi: 10.1371/journal.pntd.0010915 (PMC9668136; doi:10.1371/journal.pntd.0010915)
Supplement: S4 Fig — Incremental costs and disability-adjusted life years (DALYs) averted of full access to snake antivenom in each ASEAN country based on a probabilistic sensitivity analysis of 1,000 iterations are presented in dots. Willingness-to-pay thresholds of each country are presented as dash lines with corresponding color. (DOCX) [file pntd.0010915.s009.docx]

**S4 Figure** Cost-effectiveness plane of incremental costs per disability-adjusted life year (DALY) averted of improving access to snake antivenom in ASEAN countries

Incremental costs and disability-adjusted life years (DALYs) averted of full access to snake antivenom in each ASEAN country based on a probabilistic sensitivity analysis of 1,000 iterations are presented in dots. Willingness-to-pay thresholds of each country are presented as dash lines with corresponding color.
