# Supplementary material for: Potential economic and clinical implications of improving access to snake antivenom in five ASEAN countries: A cost-effectiveness analysis
Source: PLoS Negl Trop Dis. 2022 Nov 16;16(11):e0010915. doi: 10.1371/journal.pntd.0010915 (PMC9668136; doi:10.1371/journal.pntd.0010915)
Supplement: Attachment — Submitted filename: 1. Author response EE improving access to antivenom.docx [file pntd.0010915.s010.docx]

**AUTHOR RESPONSE LETTER**

**PNTD-D-22-00683: Potential economic and clinical implications of improving access to snake antivenom in five ASEAN countries: a cost-effectiveness analysis**

Dear Editor,

On behalf of all the authors, we would like to resubmit our manuscript entitled: “PNTD-D-22-00683: Potential economic and clinical implications of improving access to snake antivenom in five ASEAN countries: a cost-effectiveness analysis (PNTD-D-22-00588)”. The comments forwarded from the editor and reviewers are highly appreciated and contain useful suggestions. We have revised our manuscript and responded point-by-point to each issue raised by the reviewers. We do appreciate all valuable comments from the reviewers and have tried our best to incorporate them in this revised version.

All authors have contributions that meet the criteria for authorship and all authors have signed a statement attesting to authorship, disclosing all potential conflicts of interest, and releasing the copyright should the manuscript be accepted for publication. We attest that this work is original and has not been published and is not being considered for publication elsewhere, in any language.

Thank you for considering this paper for publication.

Sincerely yours,

Suthira Taychakhoonavudh, PhD

Department of Social and Administrative Pharmacy, Faculty of Pharmaceutical Sciences, Chulalongkorn University

Email: [Suthira.T@chula.ac.th](mailto:Suthira.T@chula.ac.th)

Tel: +662-218-8386-90

Nathorn Chaiyakunapruk, PhD

Department of Pharmacotherapy, College of Pharmacy, University of Utah

Email: [Nathorn.Chaiyakunapruk@utah.edu](mailto:Nathorn.Chaiyakunapruk@utah.edu)

Tel: +1.801.585.3092

**METHODS**

**Reviewer #1: The estimated logistic cost of only 5% is rather low.**

Response: Thank you for your valuable comments. The estimated logistic cost of 5% of antivenom price is based on the standard cost list of Thailand which is a recommended source for estimating health care costs for economic evaluation. However, we agreed that the logistic costs could be higher. Therefore, we have performed an additional scenario analysis of increasing the logistics costs to 10% of the antivenom price. We found that the cost-effectiveness estimates remained robust (S2 & S3 Table in the Supplement). We have elaborated on this in the Methods, Sensitivity analyses, Line 219-222, on Page 9-10, and Results, Sensitivity analyses, Line 264-267, on Page 12. Please see the changes to the manuscript below.

| **Original version** | **Revised version** |
| --- | --- |
| - | Methods, Sensitivity analyses, Line 219-222, on Page 9-10  Lastly, scenario analyses were performed by increasing the logistic costs from 5% to 10% of antivenom price because these could be higher, especially in archipelagic countries like Indonesia and Philippines. |
| Scenario analyses were presented in **S2 Table** and **S3 Table** in the **supplementary material**. Incorporating PTSD in the model and assuming that antivenom could reduce the risk of amputation found similar ICERs per death averted and ICERs per DALY averted in all countries indicating that improving access will result in cost-saving. | Results, Sensitivity analyses, Line 264-267, on Page 12  Scenario analyses were presented in **Tables S2** and **S3**. Incorporating PTSD in the model, assuming that antivenom could reduce the risk of amputation, and increasing the logistic costs of antivenom resulted in similar ICERs per death averted and ICERs per DALY averted in all countries indicating that improving access will result in cost-saving. |

**Reviewer #2: The objectives of the study are clear and well founded. In this work the authors selected five most impacted ASEAN countries for this study, including Indonesia, Philippines, Vietnam, Lao PDR, and Myanmar. However the title refers to the whole group of ASEAN countries. The title should be modified as to mention that it includes only a group of ASEAN countries, the ones having the highest load of SBE. The methodology is clearly described, and the statistical analyses are presented. There are no ethical issues concerning this study.**

Response: Thank you for your valuable comments. We agreed with your comment. We have revised our title. Please see the changes to the manuscript below.

| **Original version** | **Revised version** |
| --- | --- |
| Potential economic and clinical implications of improving access to snake antivenom in ASEAN countries: a cost-effectiveness analysis | Potential economic and clinical implications of improving access to snake antivenom in five ASEAN countries: a cost-effectiveness analysis |

**I have several comments concerning specific issues in the Methodology:**

**The disabilities included in the model were digit and lib amputations. Although these disabilities are probably the most significant ones, studies in this topic have identified other types of physical and psychological disabilities which may have a great impact in the quality of life of affected people. In particular, victims of Daboia sp often develop chronic kidney failure, which involves high costs of treatment. This would be a relevant parameter to consider, and there is information in the literature on the incidence of this problem and the incidence of Daboia sp cases, at least in some of these countries or in neighbouring countries. Thus, the disability analysis could be expanded. In the section of limitations of the study, the authors indicated that “other disabilities of snakebite envenoming such as blindness, malignant ulcers, and pregnancy loss were not included due to lack of empirical evidence”. However, in the case of chronic renal failure, which involves high costs from the medical system, I believe there is published information which can provide valuable inputs to enrich the analysis.**

Response: We agreed with your comment on the importance of incorporating broader scope of disabilities of snakebite envenoming. Renal consequences due to snakebite envenoming were brought up when we discussed with local experts. It was found that Russell’s viper bite caused acute kidney injury. However, information on chronic kidney disease following Russell’s viper bite in ASEAN was not documented because patients were lost to follow-up after they were discharged. Thus, we have decided to keep our estimate conservative without incorporating renal consequences in the model while acknowledging this limitation. We have elaborated on this in the Discussion, Limitations, Line 360-367, on Page 17. Please see the changes to the manuscript below.

| **Original version** | **Revised version** |
| --- | --- |
| Secondly, other disabilities of snakebite envenoming such as blindness, malignant ulcers, and pregnancy loss were not included due to lack of empirical evidence.^7^ | Thirdly, other disabilities of snakebite envenoming such as blindness, malignant ulcers, and pregnancy loss were not included due to lack of empirical evidence.^7^ Chronic kidney disease due to Russell’s viper (*Daboia russelii*) bite was not included in our study. It was found that Russell’s viper bite caused acute kidney injury. However, information on chronic kidney disease following Russell’s viper bite in ASEAN was not documented because patients were lost to follow-up after they were discharged.^39-41^ This emphasizes the importance of funding future studies in ASEAN to evaluate all relevant consequences and disabilities and associated costs of snakebite to allow better estimation of the cost-effectiveness of improving access to antivenom. |

**The authors state that “antivenom effectiveness was based on a study in Nigeria which found 2.33 folds (95% confidence interval; 1.26-4.06) increased risk of death in indicated victims who were not treated with antivenom compared to those treated with antivenom”. Extrapolation of this assumption from Nigeria to South East Asia may have limitations. In Nigeria most snakebites are inflicted by snakes of the genus Echis, for which antivenoms in general have higher efficacy. In the case of Asia, many snakebites are inflicted by species of Naja and Bungarus, which may induce severe neurotoxicity of rapid onset, or by Daboia sp, which inflict highly complicated cases involving severe renal injury and other consequences. Hence, this extrapolation has limitations. It would be more pertinent to find such numbers in studies carried out in India, for example, where the types of snakes are similar to the ones in ASEAN countries.**

Response: We agreed with your comment to acknowledge the limitation of the assumption of antivenom effectiveness based on a study in Nigeria. However, to the best of our knowledge, there is no such information in India. We have acknowledged this limitation in the Discussion, Limitations, Line 351-356, on Page 17. Please see the changes to the manuscript below.

| **Original version** | **Revised version** |
| --- | --- |
| Firstly, all input parameters used in the model were derived from the available published literature, local data, and expert opinion when data were not available. These input parameters carried inherent uncertainty. Nevertheless, our sensitivity analyses showed robust conclusions of the model. | Firstly, all input parameters used in the model were derived from the available published literature, local data, and expert opinion when data were not available. These input parameters carried inherent uncertainty. We assumed antivenom effectiveness based on a study in Nigeria.^11^ Most snakebites In Nigeria are inflicted by snakes of the genus *Echis*, for which antivenoms, in general, have high effectiveness.^7^ This assumption may have a limitation as snakes in Nigeria are different from ASEAN. Nevertheless, our sensitivity analyses showed robust conclusions of the model. |

Moreover, we performed threshold analyses to estimate the lowest level of antivenom effectiveness that would result in the “not cost-saving” situation where ICER is equal to zero as shown in S4 Table below. We have elaborated on this in the Methods, Sensitivity analyses, Line 224-226, on Page 10, in the Results, Sensitivity analyses, Line 273-277, on Page 12, and in the Discussion, Line 314-318, on Page 15. Please see the changes to the manuscript below.

**S4 Table** Threshold analyses of antivenom effectiveness and costs of antivenom treatment resulted in an incremental cost-effectiveness ratio of 0

| Parameter | Indonesia | Philippines | Vietnam | Lao PDR | Myanmar |
| --- | --- | --- | --- | --- | --- |
| Antivenom effectiveness, Risk ratio of death | | | | | |
| Base-case value | 2.33 | 2.33 | 2.33 | 2.33 | 2.33 |
| Threshold value resulting in ICER of 0 | 1.01 | 0.44 | 0.73 | 0.35 | 1.19 |
| Costs of antivenom treatment, USD |  |  |  |  |  |
| Base-case value | 773 | 312 | 68 | 202 | 326 |
| Threshold value resulting in ICER of 0 | 22,985 | 46,054 | 5,624 | 22,365 | 3,083 |
| Ratio of threshold value to base-case value | 30 | 149 | 87 | 113 | 9 |

| **Original version** | **Revised version** |
| --- | --- |
| - | Methods, Sensitivity analyses, Line 224-226, on Page 10  Threshold sensitivity analyses were performed to estimate the lowest level of antivenom effectiveness and the highest level of costs of antivenom treatment that would result in the “not cost-saving” situation where ICER is equal to zero but still considered cost-effective. |
| - | Results, Sensitivity analyses, Line 273-277, on Page 12  Threshold analyses of antivenom effectiveness and costs of antivenom treatment resulted in an ICER of 0 are shown in **S4 Table**. The lowest level of antivenom effectiveness, presented as a risk ratio of death in indicated victims who were not treated with antivenom compared to those treated with antivenom, was ranging from 0.35 in Lao PDR to 1.19 in Myanmar. The highest level of costs of antivenom treatment was ranging from 9 to 149 times the base-case value in Myanmar and Lao PDR, respectively. |
| - | Discussion, Line 314-318, on Page 15  We performed a series of sensitivity analyses and found that the conclusion of our study remained robust. Threshold analyses found that the antivenom effectiveness could be as low as 0.35 to 1.19, and the costs of antivenom treatment could increase as high as 9 to 149 times of the base-case value to render improving access to antivenom no longer a cost-saving strategy. This emphasizes the cost-effectiveness of improving access to antivenom. |

**It is stated that “In the full access scenario, all snakebite victims who firstly sought traditional healers when access is now full are assumed to switch to conventional treatment”. This assumption is valid for the purpose of the study. However, an additional factor is the time needed to reach health facilities, which may largely differ depending on the country and the region, the development of the public health system and circumstances such as the quality of the roads and climate variables, as has been studied for the case of SBE in Nepal. I realize these factors are difficult to introduce in the model used, but at least should be acknowledged in the Discussionm because the time to reach medical attention is directly related to the prognosis of the cases, both in terms of mortality and long term disability..**

Response: We agreed with your comment. We have acknowledged this limitation in the Discussion, Limitations, Line 365-369, on Page 17. Please see the changes to the manuscript below.

| **Original version** | **Revised version** |
| --- | --- |
| Thirdly, we assumed that in all snakebite victims in full access would eventually seek conventional treatment. However, this might not be possible because not all victims could timely access to healthcare facilities especially those who lived in the farthest rural areas. This is especially important as timely access to healthcare facilities is related to the prognosis of the victims, both in terms of mortality and long-term disability.^42,43^ | Thirdly, we assumed that all snakebite victims in full access would eventually seek conventional treatment. However, this might not be possible because not all victims could have timely access to healthcare facilities, especially those who lived in the farthest rural areas. This is especially important as timely access to healthcare facilities is related to the prognosis of the victims, both in terms of mortality and long-term disability.^42,43^ |

**In estimating the cost of antivenom treatment, the authors have probably based their analyses in the current cost of antivenom in these countries (which I believe vary a lot) and on the usual number of antivenom vials used in a single treatment. However, if the study is based on the idea that the antivenoms to be used are geographically appropriate and effective, both the cost per vial and the number of vials needed for a treatment may vary as compared to the current situation. How are these parameters considered in the analysis?**

Response: Costs of antivenom treatment were estimated based on the average dose of antivenom vials used in the treatment of snakebite envenoming with the consideration of different types of snakes and antivenoms in each country. We have elaborated on this in the Methods, Costs, Line 177-179, on Page 8. Please see the changes to the manuscript below.

| **Original version** | **Revised version** |
| --- | --- |
| Direct costs included costs of hospitalization, antivenom treatment, antivenom logistics, adverse drug reaction management, amputation, transportation, and additional food for victims and their relatives. | Direct costs included costs of hospitalization, antivenom treatment, antivenom logistics, adverse drug reaction management, amputation, transportation, and additional food for victims and their relatives. Costs of antivenom treatment were estimated based on the average dose of antivenom vials used in the treatment of snakebite envenoming with the consideration of different types of snakes and antivenoms in each country. |

Moreover, we performed threshold analyses to estimate the lowest level of antivenom effectiveness that would result in the “not cost-saving” situation where ICER is equal to zero as shown in S4 Table below. We have elaborated on this in the Methods, Sensitivity analyses, Line 224-226, on Page 10, in the Results, Sensitivity analyses, Line 273-277, on Page 12, and in the Discussion, Line 314-318, on Page 15. Please see the changes to the manuscript below.

**S4 Table** Threshold analyses of antivenom effectiveness and costs of antivenom treatment resulted in an incremental cost-effectiveness ratio of 0

| Parameter | Indonesia | Philippines | Vietnam | Lao PDR | Myanmar |
| --- | --- | --- | --- | --- | --- |
| Antivenom effectiveness, Risk ratio of death | | | | | |
| Base-case value | 2.33 | 2.33 | 2.33 | 2.33 | 2.33 |
| Threshold value resulting in ICER of 0 | 1.01 | 0.44 | 0.73 | 0.35 | 1.19 |
| Costs of antivenom treatment, USD |  |  |  |  |  |
| Base-case value | 773 | 312 | 68 | 202 | 326 |
| Threshold value resulting in ICER of 0 | 22,985 | 46,054 | 5,624 | 22,365 | 3,083 |
| Ratio of threshold value to base-case value | 30 | 149 | 87 | 113 | 9 |

| **Original version** | **Revised version** |
| --- | --- |
| - | Methods, Sensitivity analyses, Line 224-226, on Page 10  Threshold sensitivity analyses were performed to estimate the lowest level of antivenom effectiveness and the highest level of costs of antivenom treatment that would result in the “not cost-saving” situation where ICER is equal to zero but still considered cost-effective. |
| - | Results, Sensitivity analyses, Line 273-277, on Page 12  Threshold analyses of antivenom effectiveness and costs of antivenom treatment resulted in an ICER of 0 are shown in **S4 Table**. The lowest level of antivenom effectiveness, presented as a risk ratio of death in indicated victims who were not treated with antivenom compared to those treated with antivenom, was ranging from 0.35 in Lao PDR to 1.19 in Myanmar. The highest level of costs of antivenom treatment was ranging from 9 to 149 times the base-case value in Myanmar and Lao PDR, respectively. |
| - | Discussion, Line 314-318, on Page 15  We performed a series of sensitivity analyses and found that the conclusion of our study remained robust. Threshold analyses found that the antivenom effectiveness could be as low as 0.35 to 1.19, and the costs of antivenom treatment could increase as high as 9 to 149 times of the base-case value to render improving access to antivenom no longer a cost-saving strategy. This emphasizes the cost-effectiveness of improving access to antivenom. |

**RESULTS**

**Reviewer #1: It is interesting to know the Budget Impact that each country government (or another payer) needs to pay per year for this antivenom access.**
Response: Thank you for your valuable comments. Table 1 shows the incremental direct costs of improving access to antivenom which ranged from an incremental of 1 million USD in Lao PDR to 50 million USD in Indonesia. A comprehensive budget impact analysis is, however, considered out of the scope of our analysis.

**Reviewer #2: Results are presented in a clear manner and the analysis matches the analysis plan described in the methodology. The figures and tables are good and of enough quality and clarity.**
Response: Thank you for your time reviewing our manuscript.

**CONCLUSION**

**Reviewer #1: Yes**
Response: Thank you for your time reviewing our manuscript.

**Reviewer #2: The main cionclusions are supported by the data presented. However, as indicated above in the comments to the methodology, there are extrapolations in this study which may affect the conclusions. The authors acknowledge some limitations of the study, but should expand this by including other aspects mentioned above.**
Response: Thank you for your valuable comments. We have revised the manuscript to address your suggestions.

**Since only five countries of ASEAN countries were included in the analysis, it is recommended that the authors acknowledge this limitation and include some considerations on whether this could apply to the whole region or not.**
Response: We have explained why we conducted the analysis in five countries in the Methods, Setting, Line 128-135, on Page 6-7. *“We selected the five most impacted ASEAN countries for this study, including Indonesia, Philippines, Vietnam, Lao PDR, and Myanmar because the previous estimates in these countries found that only 4-64% of victims who were indicated for antivenoms were treated with antivenoms.^5^*

*Malaysia and Thailand were not selected because more than 90% of victims in these countries who were indicated for antivenoms were treated with antivenoms.^11^ Brunei Darussalam and Singapore were not selected because snakebite rarely occurs and/or exact data were lacking.^1^ Cambodia was not selected due to a lack of information and key informants, although it is one of the countries with a high incidence of snakebites.^1^”*

We addressed the limitation of not including Cambodia in the Discussion, Line 351-354, on Page 16. Please see the changes to the manuscript below.

| **Original version** | **Revised version** |
| --- | --- |
| - | Firstly, although Cambodia is one of the countries with snakebite victims, Cambodia was not included in this study because of a lack of published literature and key informants. However, given our findings in five ASEAN countries, improving access to antivenom would be highly cost-saving as well. |

**It would be relevant to discuss possible differences between the countries analyzed regarding the snake species that inflict the majority of accidents in each country, the type of clinical picture that they induce and the possible types of sequelae. For example, the incidence of amputation may be higher in countries where bites are predominantly inflicted by snakes having necrotizing effects, such as viperid species and cytotoxic cobras. In contrast, in a country where most cases are inflicted by kraits (Bungarus sp), the likelihood of necrosis and amputation is low, but the likelihood of severe neurotoxic envenoming leading to death is high. On the other hand, in a country where many bites are inflicted by Daboia sp, the possibilities of chronic kidney failure is high. The analysis of these issues, or at least their Discussion, would be enriched with the description of these possible national differences.** 
Response: We agreed with your comment. We have elaborated on this in the Discussion, Line 311-318, on Page 15. Please see the changes to the manuscript below.

| **Original version** | **Revised version** |
| --- | --- |
| - | Mortality and disabilities of snakebite envenoming in each country differ in regards to differences in the toxicity and lethality of the snakes.^5,9^ Nevertheless, improving access to antivenom was found to be cost-saving in all five ASEAN countries regardless of differences in snakes causing the snakebite envenoming and baseline level of access to antivenom. We performed a series of sensitivity analyses and found that the conclusion of our study remained robust. Threshold analyses found that the antivenom effectiveness could be as low as 0.35 to 1.19, and the costs of antivenom treatment could increase as high as 9 to 149 times of the base-case value to render improving access to antivenom no longer a cost-saving strategy. This emphasizes the cost-effectiveness of improving access to antivenom. |

We agreed with your comment on the importance of incorporating broader scope of disabilities of snakebite envenoming. Renal consequences due to snakebite envenoming were brought up when we discussed with local experts. It was found that Russell’s viper bite caused acute kidney injury. However, information on chronic kidney disease following Russell’s viper bite in ASEAN was not documented because patients were lost to follow-up after they were discharged. Thus, we have decided to keep our estimate conservative without incorporating renal consequences in the model while acknowledging this limitation. We have elaborated on this in the Discussion, Limitations, Line 360-367, on Page 17. Please see the changes to the manuscript below.

| **Original version** | **Revised version** |
| --- | --- |
| Secondly, other disabilities of snakebite envenoming such as blindness, malignant ulcers, and pregnancy loss were not included due to lack of empirical evidence.^7^ | Thirdly, other disabilities of snakebite envenoming such as blindness, malignant ulcers, and pregnancy loss were not included due to lack of empirical evidence.^7^ Chronic kidney disease due to Russell’s viper (*Daboia russelii*) bite was not included in our study. It was found that Russell’s viper bite caused acute kidney injury. However, information on chronic kidney disease following Russell’s viper bite in ASEAN was not documented because patients were lost to follow-up after they were discharged.^39-41^ This emphasizes the importance of funding future studies in ASEAN to evaluate all relevant consequences and disabilities and associated costs of snakebite to allow better estimation of the cost-effectiveness of improving access to antivenom. |

**As indicated above in the comments on the methodology, using the data on the reduction of the risk of death from a study in Nigeria, and extrapolating it to ASEAN countries has important limitations that need to be acknowledged. It would be relevant to check whether there is information on mortality due to SBE in India between people treated with antivenom and those who were not treated. At least some information on this regard may indicate whether the extrapolation from data of Nigeria is a valid approximation.**
Response: We agreed with your comment to acknowledge the limitation of the assumption of antivenom effectiveness based on a study in Nigeria. However, to the best of our knowledge, there is no such information in India. We have acknowledged this limitation in the Discussion, Limitations, Line 354-360, on Page 17. Please see the changes to the manuscript below.

| **Original version** | **Revised version** |
| --- | --- |
| Firstly, all input parameters used in the model were derived from the available published literature, local data, and expert opinion when data were not available. These input parameters carried inherent uncertainty. Nevertheless, our sensitivity analyses showed robust conclusions of the model. | Secondly, all input parameters used in the model were derived from the available published literature, local data, and expert opinion when data were not available. These input parameters carried inherent uncertainty. We assumed antivenom effectiveness based on a study in Nigeria.^11^ Most snakebites In Nigeria are inflicted by snakes of the genus *Echis*, for which antivenoms, in general, have high effectiveness.^7^ This assumption may have a limitation as snakes in Nigeria are different from ASEAN. Nevertheless, our sensitivity analyses showed robust conclusions of the model. We strongly emphasized the need to conduct comprehensive research to estimate the true burden of snakebite in ASEAN. |

Moreover, we performed threshold analyses to estimate the lowest level of antivenom effectiveness that would result in the “not cost-saving” situation where ICER is equal to zero as shown in S4 Table below. We have elaborated on this in the Methods, Sensitivity analyses, Line 224-226, on Page 10, in the Results, Sensitivity analyses, Line 273-277, on Page 12, and in the Discussion, Line 314-318, on Page 15. Please see the changes to the manuscript below.

**S4 Table** Threshold analyses of antivenom effectiveness and costs of antivenom treatment resulted in an incremental cost-effectiveness ratio of 0

| Parameter | Indonesia | Philippines | Vietnam | Lao PDR | Myanmar |
| --- | --- | --- | --- | --- | --- |
| Antivenom effectiveness, Risk ratio of death | | | | | |
| Base-case value | 2.33 | 2.33 | 2.33 | 2.33 | 2.33 |
| Threshold value resulting in ICER of 0 | 1.01 | 0.44 | 0.73 | 0.35 | 1.19 |
| Costs of antivenom treatment, USD |  |  |  |  |  |
| Base-case value | 773 | 312 | 68 | 202 | 326 |
| Threshold value resulting in ICER of 0 | 22,985 | 46,054 | 5,624 | 22,365 | 3,083 |
| Ratio of threshold value to base-case value | 30 | 149 | 87 | 113 | 9 |

| **Original version** | **Revised version** |
| --- | --- |
| - | Methods, Sensitivity analyses, Line 224-226, on Page 10  Threshold sensitivity analyses were performed to estimate the lowest level of antivenom effectiveness and the highest level of costs of antivenom treatment that would result in the “not cost-saving” situation where ICER is equal to zero but still considered cost-effective. |
| - | Results, Sensitivity analyses, Line 273-277, on Page 12  Threshold analyses of antivenom effectiveness and costs of antivenom treatment resulted in an ICER of 0 are shown in **S4 Table**. The lowest level of antivenom effectiveness, presented as a risk ratio of death in indicated victims who were not treated with antivenom compared to those treated with antivenom, was ranging from 0.35 in Lao PDR to 1.19 in Myanmar. The highest level of costs of antivenom treatment was ranging from 9 to 149 times the base-case value in Myanmar and Lao PDR, respectively. |
| - | Discussion, Line 314-318, on Page 15  We performed a series of sensitivity analyses and found that the conclusion of our study remained robust. Threshold analyses found that the antivenom effectiveness could be as low as 0.35 to 1.19, and the costs of antivenom treatment could increase as high as 9 to 149 times of the base-case value to render improving access to antivenom no longer a cost-saving strategy. This emphasizes the cost-effectiveness of improving access to antivenom. |

**When acknowledging the limitation of including only amputations as disabilities, the authors did not mention the problem of chronic renal failure that often occurs in victims of Daboia sp. This needs to be discussed as well.**
Response: We agreed with your comment on the importance of incorporating broader scope of disabilities of snakebite envenoming. Renal consequences due to snakebite envenoming were brought up when we discussed with local experts. It was found that Russell’s viper bite caused acute kidney injury. However, information on chronic kidney disease following Russell’s viper bite in ASEAN was not documented because patients were lost to follow-up after they were discharged. Thus, we have decided to keep our estimate conservative without incorporating renal consequences in the model while acknowledging this limitation. We have elaborated on this in the Discussion, Limitations, Line 360-367, on Page 17. Please see the changes to the manuscript below.

| **Original version** | **Revised version** |
| --- | --- |
| Secondly, other disabilities of snakebite envenoming such as blindness, malignant ulcers, and pregnancy loss were not included due to lack of empirical evidence.^7^ | Thirdly, other disabilities of snakebite envenoming such as blindness, malignant ulcers, and pregnancy loss were not included due to lack of empirical evidence.^7^ Chronic kidney disease due to Russell’s viper (*Daboia russelii*) bite was not included in our study. It was found that Russell’s viper bite caused acute kidney injury. However, information on chronic kidney disease following Russell’s viper bite in ASEAN was not documented because patients were lost to follow-up after they were discharged.^39-41^ This emphasizes the importance of funding future studies in ASEAN to evaluate all relevant consequences and disabilities and associated costs of snakebite to allow better estimation of the cost-effectiveness of improving access to antivenom. |

**EDITORIAL AND DATA PRESENTATION MODIFICATIONS?**

**Reviewer #1: (No Response)**

Response: Thank you for your time reviewing our manuscript.

**Reviewer #2: The manuscript is well written and presented, I do not have editorial suggestions.**

Response: Thank you for your time reviewing our manuscript.

**SUMMARY AND GENERAL COMMENTS
Reviewer #1: The authors performed a cost-effectiveness analysis of snake antivenom in ASEAN countries. By saving lives and preventing disabilities, the full antivenom access is cost-saving. The result is important for the health care policy changes in these countries.  
1. It is interesting to know the Budget Impact that each country government (or another payer) needs to pay per year for this antivenom access.**

Response: Thank you for your valuable comments. Table 1 shows the incremental direct costs of improving access to antivenom which ranged from an incremental of 1 million USD in Lao PDR to 50 million USD in Indonesia. A comprehensive budget impact analysis is, however, considered out of the scope of our analysis.

**2. It is interesting to know the reasons why the antivenom access is limited in each country. Are there major problems in antivenom production and/or logistics? The solution may not be simple by just increasing payment for the antivenom costs.**

Response: We agreed with you that the problems in each country are different. We previously assessed situation of access antivenom in ASEAN countries in another publication *(BMJ Global Health. 2022;0:e007639)*. We have discussed the potential solutions for ASEAN in the Discussion, Paragraph 1, Line 339-344, on Page 16. We have explained that:

*“We previously discussed the potential opportunities to improve access to antivenom in ASEAN that included accurate estimation of antivenom demand, rigorous regulations of antivenom, strengthening supply chain system, raising public awareness about the importance of treating snakebite envenoming by healthcare professionals, strengthening health system to ensure appropriate snakebite management and rational use of antivenoms, and expanding collaboration of local and international stakeholders to better improve access to snake antivenom for victims in the region.^9^”*

**3. The problem in each country may be different. Can they produce antivenom by themselves? The antivenom distribution in countries comprising numerous islands, i.e., Philippines and Indonesia, may be difficult. The estimated logistic cost of only 5% is rather low.**

Response: We agreed with you that the problems in each country are different. We previously assessed situation of access antivenom in ASEAN countries in another publication *(BMJ Global Health. 2022;0:e007639)*. We have elaborated on this in the Discussion, Paragraph 1, Line 296-301, on Page 15. Please see the changes to the manuscript below.

| **Original version** | **Revised version** |
| --- | --- |
| We used a decision analytic model with input parameters from various sources including published literature, local sources, and expert opinion. We did not propose specific strategies to improve access to snake antivenoms. Rather, we demonstrated the potential economic and clinical impact when all victims with snakebite envenoming in ASEAN countries were treated with geographically appropriate antivenoms. | We used a decision analytic model with input parameters from various sources including published literature, local sources, and expert opinion. We did not propose specific strategies to improve access to snake antivenoms as each country has different problems which require different strategies and policies to address them.^9^ Rather, we demonstrated the potential economic and clinical impact when all victims with snakebite envenoming in ASEAN countries were treated with geographically appropriate antivenoms. |

The estimated logistic cost of 5% of antivenom price is based on the standard cost list of Thailand which is a good source for estimating costs. However, we agreed that the logistic costs could be higher. Therefore, we have performed an additional scenario analysis of increasing the logistics costs to 10% of the antivenom price. We found that the cost-effectiveness estimates remained robust (S2 & S3 Table in the Supplement). We have elaborated on this in the Methods, Sensitivity analyses, Line 219-222, on Page 9-10, and Results, Sensitivity analyses, Line 264-267, on Page 12. Please see the changes to the manuscript below.

| **Original version** | **Revised version** |
| --- | --- |
| - | Methods, Sensitivity analyses, Line 219-222, on Page 9-10  Lastly, scenario analyses were performed by increasing the logistic costs from 5% to 10% of antivenom price because these could be higher, especially in archipelagic countries like Indonesia and Philippines. |
| Scenario analyses were presented in **S2 Table** and **S3 Table** in the **supplementary material**. Incorporating PTSD in the model and assuming that antivenom could reduce the risk of amputation found similar ICERs per death averted and ICERs per DALY averted in all countries indicating that improving access will result in cost-saving. | Results, Sensitivity analyses, Line 264-267, on Page 12  Scenario analyses were presented in **Tables S2** and **S3**. Incorporating PTSD in the model, assuming that antivenom could reduce the risk of amputation, and increasing the logistic costs of antivenom resulted in similar ICERs per death averted and ICERs per DALY averted in all countries indicating that improving access will result in cost-saving. |

**Reviewer #2: This study aims to assess the cost-effectiveness of improving access to snake antivenom from current level to full access in ASEAN countries, built on a previous study in which the authors evaluated the burden of SBE in terms of number of cases, mortality and cost. As such, it is a relevant contribution to the field of SBE since this type of analysis is scarce and badly needed. These studies are difficult in part because of the lack of accurate information to feed the models used. Regardless of these limitations, the study clearly demonstrates the high impact that adequate access and use of effective and safe antivenoms would have in ASEAN countries. The authors should consider several issues mentioned in my comments to the methodology since they may contribute to further expanding and improving their analysis. I think tha inclusion of more analyses, along the lines indicated in my comments to the methods, would enrich this work.**
Response: Thank you for your valuable comments. We have revised the manuscript to address your suggestions.
